# Vaccine inequity: a threat to Africa’s recovery from COVID-19

**DOI:** 10.1186/s41182-023-00564-2

**Published:** 2023-12-19

**Authors:** Calvin R. Wei, Samuel Kamande, Godwin C. Lang’at

**Affiliations:** 1 Department of Research and Development, Shing Huei Group, Taipei, Taiwan; 2 School of Medicine, Maseno University, Maseno, Kenya; 3 Department of Public and Global Health, University of Nairobi, Nairobi, Kenya

**Keywords:** SARS-CoV-2, COVID-19, COVID-19 vaccine, COVAX, Vaccine hesitancy, vaccine acceptance, acceptance, Sub-Saharan Africa, Vaccine confidence, Vaccine inequity, Misinformation

## Abstract

**Background:**

Vaccine inequity is a reality facing the Sub-Saharan Africa region as vaccine nationalism from high-income countries (HICs) leads to limited access to the lifesaving vaccines needed to end the pandemic. In Africa, a significant portion of the population has yet to be vaccinated against Covid-19; however, the barriers to accessing such vaccines, including capacity challenges, still persist despite the implementation of the COVAX facility meant to support the lower- and middle-income countries (LMICs) to boost vaccination.

**Methods:**

This study involved a systemic narrative review where literature search was conducted using the NCBI’s PMC and BMC databases based on defined keywords. Three authors were involved in the literature search and consensus was applied to settle disagreements and validate the findings.

**Results:**

In this systematic narrative review, we report that vaccine nationalism remains a challenge for LMICs as HICs still hoard vaccines and even bypass COVAX to procure doses directly from the manufacturers. Factors that promote vaccine hesitancy in Africa include misinformation regarding the Covid-19 vaccine, a lack of trust in politicians and the pharmaceutical industry, and concerns about vaccine safety and efficacy. The policies implemented to enhance vaccine coverage in Africa, such as mandates, community engagement, and partnerships, all seek to promote equity of vaccination and ending Covid-19.

**Conclusion:**

Covid-19 vaccine inequity persists and contributes to prolonged pandemic in LMICs. In response, African governments have taken certain measures to enhance vaccine uptake but more needs to be done to address resistance to vaccines.

## Introduction

The COVID-19 pandemic continues to ravage the world to-date, with oscillating spikes throughout the 2023 year. The intervening measures reduced mortality and morbidity rates, but the impact on economies, freedoms of people across the globe, and human social lives was severe. Accordingly, governments resorted to vaccination as the expedient strategy to ensure the world returns to normalcy by decreasing disease transmission as well as reducing the severity and associated morbidity and mortality. In Israel, more than 10 million doses were vaccinated within 4 months, with 54% of the entire population vaccinated, and 88% of people aged 50 years or older with two doses translated to rapid declines in COVID-19 cases across all age groups to 149 cases per day by 2021, April 19 [1]. The United States (US) and the European Union (EU) have made significant strides in the post-Covid recovery; however, the African continent is still in the midst of the pandemic [2]. Africa has many countries still facing serious difficulties accessing vaccines, with 53 African countries revealing the continent is still unable to reach the targeted 70% vaccination rate from when the vaccine was rolled out between March 2021 and June 2022 [3]. However, as of 2023, Africa CDC figures indicate that 14 countries have attained > 70% vaccination [4]. The existing COVID-19 vaccine coverage disparities among low-income countries (LICs) and particularly Africa are a product of insufficient vaccine supplies, inequitable distribution, weak health systems, limited vaccine production in LICs, vaccine misconceptions, and high proportions of vaccine hesitancy [5]. In a 15-country study done in February 2021 covering the Africa Centers for Disease Control and Prevention (CDC) regions, vaccine acceptance, safety concerns, skepticism towards a COVID-19 vaccine, and misinformation were the leading challenges to vaccination in Africa [6].

The vaccination rate on the African continent represented only 25% of the total population being fully vaccinated against COVID-19 by November 2022, despite the continent accounting for 17% of the global population [7]. This stands in contrast with reports indicating that the developed nations had already secured 60% of the total SARS-CoV-2 vaccines by 2021, with even some countries already pre-ordering vaccine doses that would be sufficient to vaccinate their populations several times [8]. Vaccine access has not been the only challenge facing Africa, as during the early days of the roll outs of vaccinations, distasteful commentaries were already rife portraying the continent as the location for COVID-19 vaccine testing, which generated public or social anxiety and apprehension towards acceptance [9]. This situation created the imperative on African governments to be more transparent and provide evidence-based strategies supporting SARS-CoV-2 vaccines as well as compelling them to design equitable and effective vaccine delivery plans for the populace. The moral imperative of ensuring equitable access to vaccines during the pandemic has been guided by public health interests as well as the political and global economic welfare. Global solidarity and coordination would be required to fulfill this objective of equity, although there has been little evidence to confirm such efforts have been made. Nonetheless, there were efforts to raise financing for vaccines in Africa, through the COVID-19 African Vaccine Acquisition Task Team (AVATT) established by the then African Union (AU) chairperson President Cyril Ramaphosa of South Africa and co-chaired by the AU Commission Chairperson Moussa Faki Mohamat [10]. In the views of the World Health Organization’s (WHO) Director Tedros Ghebreyesus, vaccine access gaps have created a two-tier pandemic characterized by “vaccine apartheid” between the high-income countries (HICs) and lower- and middle-income countries (LMICs) [11]. Thus, it is the objective of this review to explore the programs and research improving equity of COVID-19 vaccination, policies related to COVID-19 vaccination, and social and behavioral science strategies supporting vaccine acceptance in Africa.

## Materials and methods

### Literature search

A systematic literature search was conducted using the National Center for Biotechnology Information’s (NCBI) PubMed Central (PMC) and BioMed Central (BMC) databases based on the following search strategy:

- PMC search string: (COVID-19 Vaccine Inequality) AND (Vaccination Policies) AND (Africa).
- BMC search string: (COVID-19 Vaccine Inequality) AND (COVID-19 Vaccine Policies) AND (COVID-19 Vaccines) AND (Acceptance) AND (Hesitancy) AND (Africa).

The bibliographies of the identified articles from the databases were also screened for relevant studies that fit the inclusion criteria and the available articles were included in the present systematic narrative review. The literature search was conducted by three authors (C.R.W., S.K., and G.C.L.) and consensus was used to settle any disagreements and to validate the findings of each article.

### Inclusion and exclusion criteria

Eligible articles met the following inclusion criteria: peer-reviewed, published, empirical studies primarily focusing on COVID-19 vaccination and discussing vaccine acceptance/hesitancy, vaccination policies, published in English, specific to Africa or including African countries, published between January 2021 and July 2023, and with no limitations on study design or type of publication. Studies referring to LMICs and COVAX were also included in this review, even when not referring specifically to Africa. The exclusion criteria were: articles not focused on COVID-19 vaccinations within the African continent and non-English articles.

## Results

### Characteristics of eligible studies

#### Characteristics of eligible studies

**Fig. 1:**
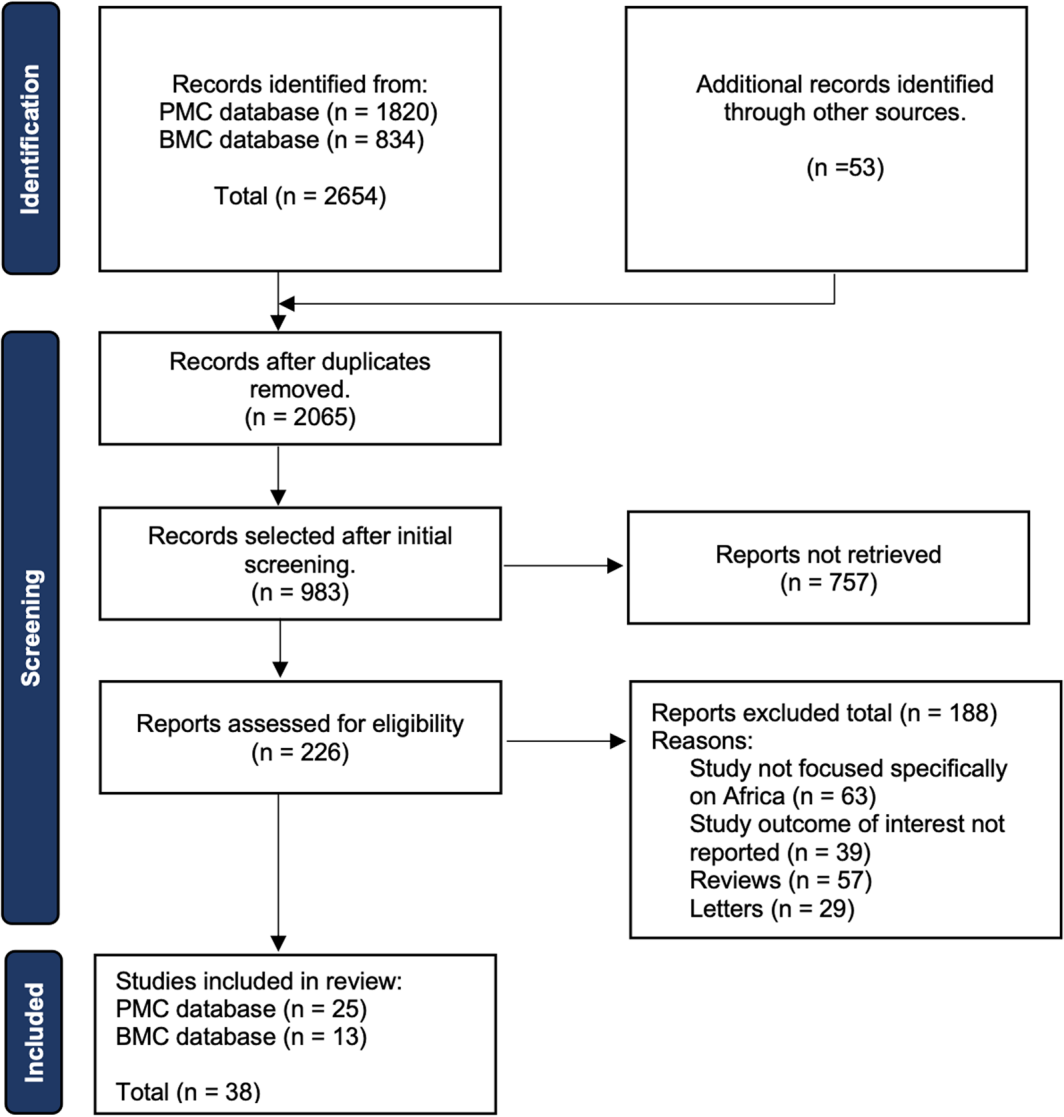
PRISMA diagram for selection of articles

The search on the PMC database yielded a total of 1820 results based on the search string “COVID-19 vaccine inequality in Africa” before any screening and identification was done. From the BMC search, a total of 834 articles were recorded based on the search string “COVID-19 vaccine policies in Africa”. After the selection, 25 articles met the inclusion criteria from PMC and 13 from BMC, making a total of 38 studies included in the present systematic review (Fig. 1).

### Equity of COVID-19 vaccination

Several studies from the search highlighted the framework of the COVID-19 Vaccines Global Access (COVAX) facility, which involved research investments and a series of international diplomatic engagements, as the path through which LMICs believed would ensure access to vaccines to manage the pandemic [12, 13]. However, with the intertwining of politics with production and distribution of COVID-19 vaccines, this framework failed to deliver the fairness and equity it was expected. Another study described the impact of donors’ and industry’s national security, diplomatic, and commercial interest pursuits that undermined dose-sharing and vaccine delivery, thereby aggravating vaccine access challenges for LMICs [14]. The vaccine “hoarding” problem by high-income countries (HICs) also emerged as part of the discussion regarding the limitations of the COVAX initiative, especially based on the facility’s inability to compete with the HICs that pursued their own bilateral agreements with manufacturers regarding the purchase of a huge portion of the available supplies of the approved vaccines [15]. In one study focused on Nigeria, emphasis was placed upon the theme of “vaccine nationalism” as a limitation to the COVAX pillar, noting that the high and upper middle-income countries prioritized their own public health needs and contributed to vaccine access inequity affecting the African continent [16]. An article focused on the Economic Community of West Africa States (ECOWAS) region reported that these HICs have the means to invest in COVID-19 vaccine research and development to bring the vaccines to market, benefiting from bilateral agreements that preserve premarket purchase commitments to vaccines still under development [17].

Africa’s access to COVID-19 vaccines has also been hamstrung by challenges arising from the initial measures taken to prevent the spread of the virus, such as social distancing and other interventions as they pushed many families into poverty, thereby hampering the capacity of African countries to finance the procurement and distribution of vaccines [18]. Even with the COVAX facility in place, many African countries have populations that exceed the number of vaccines distributed from the Global Alliance for Vaccines and Immunization (GAVI) [19]. A confusion remains regarding who to prioritize in the administration of the vaccines, just as there is a challenge associated with targeting priority regions with the highest prevalence of SARS-CoV-2 cases [20–22].

Vaccine inequality is further aggravated by the delayed vaccinations in LMICs, contributing to the emergence of new variants that will continue to prolong the pandemic as they are likely to be resistant to the already developed vaccines [23]. A key question is whether vaccination distribution is still an impactful strategy as most of the continent has missed key windows of opportunity and experienced multiple variants of concern (VOC) [24]. Limited infrastructure for storage and distribution of COVID-19 facilities in Africa also contributes to the inequality in rolling out vaccination, with most African countries even perceiving high financial cost implications whereby 60% of the funds required for successful vaccination campaigns are spent on operational costs [25]. Price variations in vaccine purchase are another challenge as evidenced by the data showing LMICs like South Africa pay more than $5 per dose for the Oxford/AstraZeneca vaccine, in contrast with the European Commission that pays $3.50 per dose [26].

Further information sourced from the WHO alongside other agencies like the African Vaccine Acquisition Trust (AVAT) alongside the Africa CDC and COVAX also underlined how the vaccine donations had a quality issue needing to be addressed [27]. In a joint statement, it was underscored how immunization benefited greatly from dose donations, although this was limited by the ad hoc nature of the donations. The little notice given and the short shelf lives of the vaccines donated translated to difficulties planning the vaccination campaigns and the efforts to increase Africa’s absorptive capacity. Consequently, the coverage rates remained low in the continent.

### Vaccine acceptance/hesitancy factors

A study on Ghana based on four online cross-sectional surveys conducted in August, 2020 (*n* = 3048), March, 2021 (*n* = 1558), June, 2021 (*n* = 1295), and February, 2022 (*n* = 424) sought to determine the levels of COVID-19 vaccine hesitancy among unvaccinated individuals [28]. This was motivated by the rationale of Africa’s ranking as the continent having the slowest vaccination rate in the world, with Ghana reporting only 27.9% (28.3% as of November 17, 2022) of the country being fully vaccinated and 37.8% receiving at least one dose as of the time of the study [29–31]. The notable reasons associated with vaccine hesitancy include lacking enough vaccine-related information, safety concerns, poor attitudes towards vaccination, mistrust in political actors, beliefs about not needing the vaccine, and concerns about side effects. The vast majority of the unvaccinated or partially vaccinated against COVID-19 tend to reside in lower-income settings in sub-Saharan Africa (SSA). With the history of political leaders failing to fulfill their political campaign promises, the populations in African countries have demonstrated a lack of trust in vaccine effectiveness as they fail to accept the words of political leaders pushing for COVID-19 [32].

A multi-country survey was also conducted to evaluate COVID-19 vaccine hesitancy among adolescents in SSA, based on computer-assisted telephone interviewing between July and December 2021 targeting Burkina Faso, Ethiopia, Ghana, Nigeria, and Tanzania and involving 2662 participants [33]. From the findings, COVID-19 vaccine hesitancy percentages were 14% in rural Kersa, 23% in rural Ibadan, 31% in rural Nouna, 32% in urban Ouagadougou, 37% in urban Addis Ababa, 45% in rural Kitampo, 65% in urban Lagos, 76% in urban Dar es Salaam, and 88% in rural Dodoma. The associated factors were perceived low necessity, concerns about vaccine safety, and concerns about the effectiveness of vaccination. In a qualitative inquiry focused on Kenya, in-depth interviews were used for data collection in urban and rural counties targeting 94 individuals from August to September 2021 [34]. Traditional media and healthcare workers were identified in this study as the common sources of vaccine information¸ and the level of vaccination hesitancy was linked to the information source for different demographics like pregnant and lactating mothers. In a systematic review to determine the factors driving vaccine hesitancy in SSA, public distrust was also common as a hindrance to the administration of COVID-19 vaccination, with the governments’ poor handling of the pandemic leading to a lack of trust from the general public, alongside the backdrop of colonialism that drives the refusal of vaccines shipped from abroad [35]. This was similar to research done in Ghana targeting 2345 adults and conducted from 23 to 28 February 2021 and whose findings showed those hesitating to get vaccinated were not well informed about the possible side effects (60%), were unsure about the clinical safety of the jab (41%), and not sure of the vaccine effectiveness in preventing COVID-19 infection (23%) [36]. The role of the media as a vaccine information source played into the willingness to get vaccinated. In a scoping review of 71 articles focused on studies conducted in Ethiopia, Kenya, Morocco, Cameroon, Botswana, Sudan, Togo, Somalia, Nigeria, Mozambique, South Africa, Zimbabwe, Uganda, Zambia, and Cote D’Ivoire, major reasons identified for vaccine hesitancy were lack of trust for pharmaceutical industries, concerns about vaccine safety and side effects, and misinformation or conflicting information from the media regarding the jab [37–40].

### COVID-19 vaccination policies

The literature search to determine the government-implemented COVID-19 vaccination policies to enhance acceptance and promote vaccine equity yielded numerous results with diverse outcomes. For instance, an ethnographic study was conducted in Ghana involving 36 respondents who participated in face-to-face in-depth interviews, among them government officials and community leaders and ten focus group discussions comprising 87 people from two administrative municipalities to explore community engagement as a vaccination policy [41]. The data collection was done from June to September 2021, with the results indicating the involvement of government institutions in engaging communities through public educational programs and radio talk shows, meetings with chiefs, community elders, religious bodies, and opinion leaders. These avenues served as outreaches to the communities and focused on vaccines and their benefits as well as dispelling myths related to the COVID-19 jab and the misinformation that was widespread, such as was the case in SSA countries like Tanzania, Kenya, Uganda, Ghana, Nigeria, Zimbabwe, and South Africa where respondents were hesitant due to misinformation about SARS-CoV-2 and vaccines [42–44].

Furthermore, several studies have examined the use of government mandates as a policy towards COVID-19 vaccine acceptance in numerous African countries. One study surveyed late-adopters presenting for vaccination a year after program initiation in Zimbabwe, with a total of 1016 adults enrolled and the results showing that 67.9% (690) approved of mandating vaccination for accessing public spaces, 67.5% (686) endorsed employer mandates, and 78.3% (796) approved of mandating COVID-19 vaccines for schools [45]. The approval of vaccine mandates was associated with perceived vaccine safety, effectiveness, and trust in regulatory processes for approving vaccines. Besides mandates, collaboration with community leaders emerged in research as another factor associated with improved vaccine uptake in Africa. For instance, one study in Western Uganda involved community dialogue meetings with district leaders in May 2021, assessing the effect on promoting vaccine uptake, whereby printed reference materials about COVID-19 and vaccines were provided to all departmental district leaders who attended [46]. There was a total of 268 attendees, 164 (61%) of whom agreed to complete the pre- and post-meeting questionnaires, and the results indicated the median COVID-19 risk perception scores changed from 3 (neutral) pre-meeting to 5 (strong agreement with being at high risk) post-meeting, with a *p* value of < 0.001. From this study, vaccine concern scores also declined, with medians changing from 4 (worried about vaccine side effects) pre-meeting to 2 (not worried) post-meeting, and a *p* value of < 0.001.

Further, a briefing paper focused on investigating the role of community-led and collaborative responses to COVID-19 in Nairobi, Kenya, applied a document analysis and qualitative data collection methods based on 30 semi-structured interviews with low-income residents and community leaders, informal workers and local organizations, non-governmental organizations (NGOs), and community health workers (CHWs) [47]. Based on the findings, residents in the low-income neighborhoods of Nairobi expressed trust in NGOs and community organizations, as well as viewing CHVs as helpful during COVID-19. The CHWs were especially instrumental in addressing the COVID-19 health crisis and raising public awareness, just as the community organizations were influential in encouraging vaccination uptake. Other studies reveal how Zambia utilized community rapid assessment to gain invaluable real-time insights concerning COVID-19 vaccination, which also allowed identification of population segments that share beliefs and motivations about vaccination, while Ghana adopted a human-centered design initiative to co-develop community-informed strategies to improve vaccination rates [48]. In a country like Rwanda, utilizing mass vaccination centers and mobile vaccination units as the government policy also increased vaccine coverage as people getting vaccinated in places like markets, bus terminals, health centers, and used mobile clinics circulating on different streets and even to homes [49].

## Discussion

**Fig. 2:**
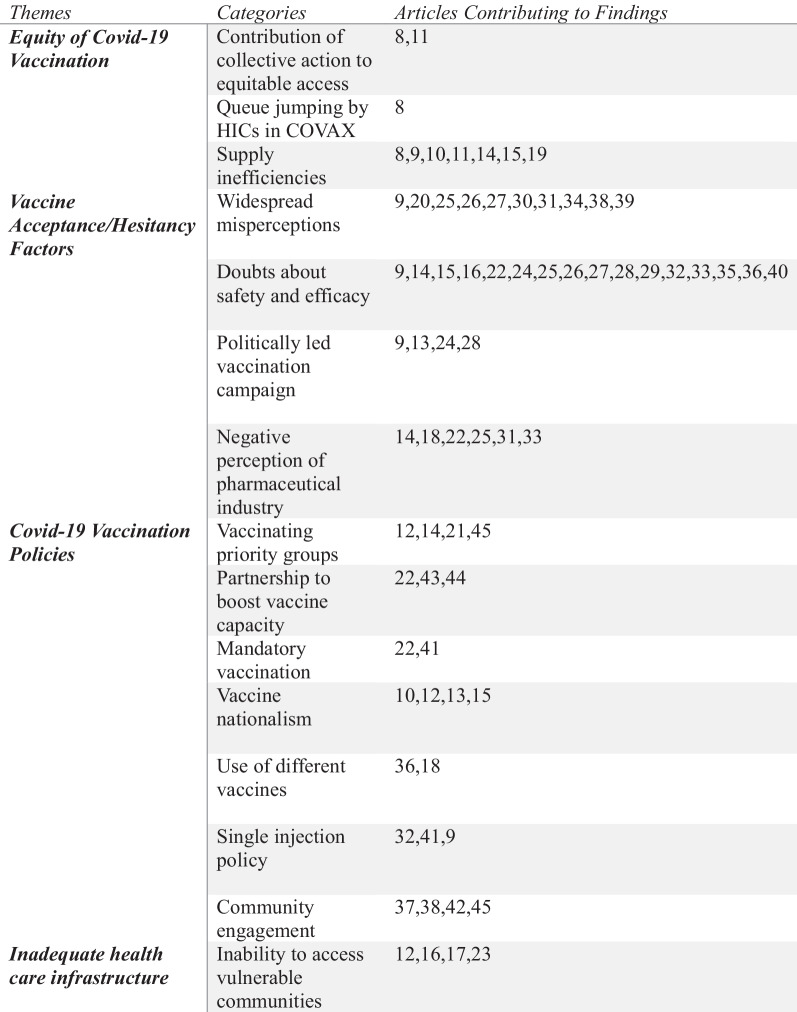
Themes derived from the various studies

The World Health Organization defines vaccine hesitancy as a ‘delay in acceptance or refusal of vaccines despite the availability of vaccination services’, with the problem being one of the greatest threats to global health since over 90% of countries worldwide have to deal with it [50]. In the African context, low COVID-19 vaccine coverage and the resultant inequality in vaccination are aggravated by vaccine hesitancy as well as vaccine nationalism and vaccine diplomacy [51]. LMICs are struggling with challenges related to inadequate vaccines as well as concerning proportions of the population who are unwilling to get vaccinated, thereby undermining the efforts to fight to end the SARS-CoV-2 pandemic. During the initial vaccine rollouts, a majority of the LMICs were unable to attain at least 10% population coverage. An ecological study evaluating the country’s economic standing against increased toll on cumulative cases and death revealed countries with the smallest economies reported first vaccination much later than larger economies [52]. Based on the study, LICs experiencing a one-day increase until the first vaccination had a 1.92% increase in cumulative cases at a 95% confidence interval (CI) compared to HICs. Similarly, there was a positive percentage increase in cumulative mortality among LMICs experiencing a 1-day increase in first vaccination, with the magnitude and direction of interaction with economic models mirroring those of cumulative cases. This is confirmation enough of the persistent COVID-19 vaccine inequalities that continue to plague LMICs such as those in sub-Saharan Africa (Fig. 2).

The efforts to achieve global vaccine equity through initiatives such as the COVAX program faced challenges related to vaccine nationalism that superseded these efforts and reveal the existence of structural inequities in accessing COVID-19 vaccines. Through a data-driven, age-stratified epidemic model to evaluate the effects of COVID-19 vaccine inequities focused on 20 LMICs from all WHO regions, it has been revealed that as of October 1, 2022, 77% of individuals in high and upper middle-income countries completed the initial vaccination course, with its equivalent in LMICs being 50% [53]. In this same study, the vaccine coverage by October 1, 2021 in HICs was more than 1 dose per person while that of lower middle-income countries were 40 doses per 100 and low-income countries stood at 3.6 doses per 100, with these inequalities further discussed in several other studies [54–58]. In HICs, challenges related to vaccine hesitancy and dose scarcity have been anticipated, with governments implementing potential solutions like mass media interventions and population groups prioritization to increase uptake. However, in LMICs, there are greater and additional challenges affecting the rates of vaccinations, with vaccine hesitancy, low resource availability, inadequate cold-chain and storage, limited finances for surveillance, and lack of coordination with a significant private healthcare sector [59]. Therefore, with these challenges and the subsequent government response, the disparities in vaccine coverage between the wealthy versus poor nations are presented clearly.

The vaccine inequality facing the African continent is difficult to solve as countries still face challenges to procure lifesaving vaccines in a context where the wealthier countries are buying vaccines directly from the manufacturers or through multilateral initiatives. The vaccination supply problems across LMICs are compounded by the behavior of HICs offering COVID-19 booster doses to the immunocompromised and vulnerable populations, as well as the entire general populations that are often regarded low-risk to SARS-CoV-2 [60]. This level of vaccine nationalism is characterized by rapid mobilization of resources to amplify booster campaigns, paying high costs to procure additional doses, and closing borders [61]. The efforts of HICs delay the end to the pandemic as variants and infections do not respect borders. Furthermore, the behaviors of the wealthier nations undermine solidarity for global health and weaken the pandemic response. These HICs have pre-ordered large numbers of doses directly from the vaccine manufacturers like BioNTech/Pfizer, in contrast to the LMICs that relied on the COVAX facility that procured doses for distribution from AstraZeneca, Novavax, and Janssen, which were slower to overcoming scale-up challenges in manufacturing that were unknown to BioNTech/Pfizer [62]. There were also allocation, affordability, and deployment challenges facing sub-Saharan Africa that were uncommon to HICs, with the wealthier nations prioritizing national access over global equity and bypassing COVAX to access the vaccines directly from the manufacturers [19, 63]. Further evidence indicates the inadequate financial resources in SSA to procure the quantity of required vaccines, the lack of financing for sustainable funding for logistics and cold chain requirements, as well as problems of delivering vaccines to remote and hard-to-reach areas where the most vulnerable reside [64].

Vaccine inequality during the COVID-19 pandemic in Africa is reflected in the form of vaccine hesitancy/acceptance factors as determined in this review. A cross-sectional study reported in rural and urban West Africa that despite the respondents being worried about getting infected with SARS-CoV-2, half of those interviewed were unsure about the safety of the vaccine, leading to their unwillingness to get vaccinated [65, 66]. Indeed, in an explanatory, sequential, mixed-methods design study carried out in Senegal between December 24, 2020 and January 16, 2021 for quantitative data collection and February 19 to March 30, 2021 for the qualitative data, it was reported that the proportion of those who refused to be vaccinated due to the belief the vaccine could endanger their health was 67.9% [67, 68]. Accordingly, poor attitudes toward the COVID-19 vaccine were significant barriers to vaccination and this was part of the challenge facing the efforts toward achieving global vaccine equity, with most individuals in LMICs worried about the safety and effectiveness of the jab [69–71]. In a scoping review focused on SSA, participants in Cameroon, Kenya, Ethiopia, and Nigeria perceived that the vaccine effectiveness is not well studied, with 61.8% uncertain about vaccine efficacy. In Kenya alone among pregnant women, 17.4% believed in the effectiveness of the vaccines compared to 29.6% in Congo and 48.1% in Zambia [72–74].

In this review, the lack of vaccine-related information was another barrier to vaccine acceptance and contributed to vaccine inequity in SSA. Following a qualitative study involving interviews with pregnant and lactating women (PLW), health workers, and policymakers in Kenya, a total of 59 participants were interviewed and the emerging themes from the results indicated uncertainty regarding PLW eligibility for COVID-19 vaccination as a matter of concern [75]. The lack of clear guidelines regarding COVID-19 vaccine use is further emphasized by the global disparities in policies, with a systematic screening of public health authorities’ websites from 224 countries indicating 176 countries had issued explicit guidance, with 38% recommending use, 28% permitting administration, 15% permitting use with qualifications, 2% not recommending but with exceptions, and 17% not recommending administration whatsoever [76]. Notably, policymakers felt that the vaccination policy was prohibitive for administering the jab, further noting the need for individual risk assessment before administration. Another study that confirms the findings of this review was done in Tanzania’s Moshi in the Northern part of the country, which involved 232 participants in a cross-sectional interview that revealed only moderate knowledge about COVID-19 as 48.3% believed SARS-CoV-2 was man made [77]. This contributed to vaccine hesitancy because of the misinformation surrounding COVID-19 and the vaccine safety and efficacy.

A lack of trust in political actors, both local and foreign, is another factor associated with vaccine hesitancy in SSA. A study analyzing data from the Kenya Rapid Response Phone Survey (RRPS) measured vaccine refusal and the associated factors, noting that distrust in the government’s response to COVID-19 was a determiner [78]. Due to the political nature of the SARS-CoV-2 vaccine, the mandatory administration in some countries as well as the lack of trust in the pharmaceutical industry also influence vaccine acceptance, with some individuals even holding religious beliefs regarding the jab as a representation of the “mark of the beast” [79]. The delayed response to the pandemic by African governments further hampered the people’s belief in their capability to manage the crisis. In Chad, 21% of participants in a survey reported lacking trust in the government as a factor related to their refusal to vaccinate [80]. Another study among healthcare workers in Cameroon and Nigeria based on a web-based cross-sectional study also determined mistrust in the pharmaceutical industries, the government, and public health regulatory authorities influenced their decision to refuse vaccination [81].

The review also evaluated the COVID-19 vaccine policies implemented in Africa, with a mixed-methods study among health workers in rural Uganda corroborating the findings by reporting to feel coerced to take the COVID-19 vaccine [82]. Mandatory policies invoked fears in the health workers, making them hesitant about their trust in policymakers. Moreover, research in Uganda and Sierra Leone confirm ensuing anxieties and fears associated with mandating vaccination for conditions of employment. Villagers in rural Gulu, Uganda perceived support for mandatory vaccination as betrayal and alignment with the interests of the state rather than the residents [83]. Community engagement is another vaccination policy to boost uptake in Africa, which was revealed in a cross-sectional study focused on Malawi COVID-19 vaccination program where over just six months of implementing the “vaccinate my village” (VMV) program, 2.3 million vaccines were administered [84]. There were other policies like mass vaccination, mobile vaccination provision, and enhancing vaccine communication to boost acceptance.

## Conclusion and recommendations

### Conclusion

COVID-19 vaccine inequity remains a challenge that continues to prolong the pandemic in LMICs. The African continent remains one such region that still lags in the rates of vaccinations as countries in Africa still rely on the COVAX facility and vaccine donations to vaccinate the population.. Based on the literature search, three common themes were discussed in the results: equity of COVID-19 vaccination, vaccine acceptance/hesitancy factors, and vaccination policies. Currently, vaccine nationalism still exists in Africa; and they lack the capacity to vaccinate the large populations because the vaccines distributed by COVAX are limited. Challenges of prioritizing vulnerable populations still remain. New variants pose more challenges as delayed vaccinations mean the pandemic lasts longer. Regarding vaccine hesitancy, factors like lack of sufficient vaccine information, safety concerns, poor attitudes toward vaccination, mistrust in the political actors and the pharmaceutical industry, and beliefs regarding the lack of need for vaccines hamper efforts. There are certain measures that have been taken by African governments to enhance uptake of vaccines, including mandates, community engagement, and community-led and collaborative efforts. However, some like mandatory vaccination increased resistance to vaccines as coercion instilled fear and anxieties in citizens who did not trust the governments’ efforts to manage the pandemic.

### Recommendations

Therefore, based on the findings of this review, it is recommended that improving COVID-19 vaccine communication will be instrumental in achieving vaccine equity in sub-Saharan Africa because people need more knowledge about the safety and efficacy of the vaccines. Further, efforts to enhance vaccine production and distribution on the continent are welcome because relying on foreign donations face numerous challenges. This will be the surest way to mitigate current incidences in COVID-19 and bring it to an ultimate rest. It is upon the AU member states to prioritize local manufacturing to enhance the continent’s self-reliance.

## Data Availability

Data will be available upon request of the corresponding author.
